# Postoperative evaluation of visual and cognitive functions following cataract surgery in patients with age-related cataracts: a prospective longitudinal study

**DOI:** 10.3389/fnins.2024.1505585

**Published:** 2025-01-03

**Authors:** Chenzhu Zhao, Xuan Li, Bolin Deng, Bingyue Shui, Lin Zhou, Zhengzheng Wu

**Affiliations:** ^1^Department of Ophthalmology, The Affiliated Hospital, Southwest Medical University, Luzhou, China; ^2^Department of Ophthalmology, Sichuan Provincial People's Hospital, Chengdu, China

**Keywords:** age-related cataracts, cataract surgery, cognitive function, visual function, mild cognitive impairment

## Abstract

**Introduction:**

Cataracts are associated with a decline in both cognitive and visual functions. This study examines postoperative changes in cognitive and visual functions in patients with age-related cataracts, focusing on the differential effects of unilateral and bilateral cataract surgeries on these functions. Additionally, the study evaluates changes in cognitive function following cataract surgery in individuals with mild cognitive impairment (MCI).

**Methods:**

A cohort of patients (n = 35, 59 eyes) aged 60 years and older (69.9 ± 7.0 years) with age-related cataracts who underwent unilateral or bilateral cataract surgery between May and June 2024 was selected. Cognitive and visual functions were evaluated preoperatively and at 1 week, 1 month, and 3 months postoperatively. Cognitive function was evaluated using the Montreal Cognitive Assessment (MoCA). Visual function was assessed using a binocular visual function testing system based on virtual reality (VR) technology, which evaluated low spatial frequency suppression, simultaneous vision, stereopsis, and perceptual eye position under 3D viewing conditions without glasses. Based on preoperative MoCA scores, patients were classified into cognitively normal and mild cognitive impairment (MCI) groups.

**Results:**

Patients with age-related cataracts demonstrated significant improvements in both cognitive and visual functions at 1 week, 1 month, and 3 months postoperatively, compared to preoperative assessments (*p* < 0.05). Specifically, both the bilateral surgery group and the MCI group exhibited substantial improvements in cognitive function at these time points (*p* < 0.05). Additionally, the bilateral surgery group outperformed the unilateral surgery group in cognitive function throughout the follow-up period (*p* < 0.05). In terms of visual function, the bilateral surgery group showed significant improvements in low spatial frequency suppression, simultaneous vision, and stereopsis at 1 week, 1 month, and 3 months postoperatively, compared to preoperative measurements (*p* < 0.05).

**Conclusion:**

Both cognitive and visual functions significantly improved after cataract surgery. Bilateral cataract surgery is more effective in increasing the cognitive functions than unilateral surgery. Additionally, cataract surgery plays a critical role in facilitating cognitive recovery in patients with mild cognitive impairment (MCI).

## 1 Introduction

Cataracts are one of the commonest causes of vision loss, primarily characterized by lens opacity (Asbell et al., 2005). The primary symptom of cataracts is impaired vision; however, patients may also experience symptoms such as glare, monocular diplopia, myopic shift, and change in color vision (Delbarre and Froussart-Maille, 2020). Cataracts are primarily age-related disorder, and age-related cataracts are the most common type in adults (Liu et al., 2017). Globally, the prevalence of cataracts in individuals aged 60 and above reaches an alarming 88.17% (Hashemi et al., 2020). Previous studies have shown a strong association between cataracts and an increased risk of cognitive impairment (Xiong et al., 2023; Wang L. et al., 2024). Cataract surgery, as a highly effective treatment, not only restores vision but also reduces the risk of cognitive impairment and slows the rate of cognitive decline (Tamura et al., 2004; Ma et al., 2023; Lee et al., 2022; Maharani et al., 2018; Thompson and Lakhani, 2015). Furthermore, cataract surgery can result in both structural and functional improvements in brain regions associated with vision and cognition (Lin et al., 2018).

Dementia is a syndrome characterized by a significant decline in cognitive abilities, resulting in impairments in occupational, familial, or social functioning (Gale et al., 2018). According to the World Health Organization (WHO), over 55 million people worldwide are living with dementia, with 10 million new cases diagnosed annually (World Health Organization, 2021). However, some studies suggested that sensory rehabilitation interventions may reduce the risk of dementia (Chen et al., 2021; Chen et al., 2017; Kuo et al., 2021). Mild cognitive impairment (MCI) represents an intermediate cognitive state between normal aging and dementia (Ritchie and Ritchie, 2012). The global prevalence of MCI in adults aged 50 years and older is 15.56%, with higher rates observed in older compared to younger cohorts (Bai et al., 2022). MCI is considered a critical “window” for intervention, potentially delaying progression to dementia (Anderson, 2019). Approximately 20–30% of patients are expected to recover normal cognitive function within 5 years (Jia et al., 2021). Notably, maintaining good vision is critical for facilitating cognitive recovery in patients with MCI (Sachdev et al., 2013).

Cataracts and cognitive impairment are strongly associated with aging (Dintica and Yaffe, 2022; Liu et al., 2017; Bai et al., 2022). As the population continues to age, the number of patients with both cataracts and cognitive impairment is expected to rise. Despite cataract surgery has demonstrated considerable benefits for cognitive improvement, research on the differential effects of unilateral versus bilateral surgery on cognitive function remains limited. Furthermore, studies investigating postoperative cognitive changes in cataract patients with mild cognitive impairment (MCI) are limited. Previous research on the visual outcomes of cataract surgery has predominantly focused on postoperative visual acuity and visual quality, including metrics such as aberrations, modulation transfer function, and Strehl ratio (Garzón et al., 2022; Zhong et al., 2022), with limited attention has been given to the assessment of visual function. Vision formation requires complex coordination between the eyes and the brain, encompassing the acquisition, processing, and interpretation of visual information. When external light passes through the eye’s refractive system and reaches the brain, it activates two primary visual processing pathways: the ventral stream and the dorsal stream (Ungerleider and Mishkin, 1982). In the ventral stream, information flows from the primary visual cortex to the inferotemporal cortex, which plays a major role in the perceptual identification of objects. In the dorsal stream, information ascends from the primary visual cortex to the posterior parietal region, where it processes motion and spatial aspects of vision (Goodale and Milner, 1992). Advances in brain-vision science have revealed that visual dysfunctions, such as visual–spatial processing abnormalities or object recognition deficits, are strongly associated with disruptions in these two visual processing pathways (Bennett et al., 2020).

This study aims to investigate the postoperative changes in both cognitive function and visual function in patients with age-related cataracts. Additionally, the study compares the effects of unilateral versus bilateral cataract surgery on cognitive and visual function, and analyzing the postoperative cognitive changes in patients with normal cognition as well as those with MCI.

## 2 Materials and methods

### 2.1 Participants

This study is a prospective, observational investigation. Between May and June 2024, 35 elderly patients (59 eyes), aged 60 years and older, with bilateral cataracts were recruited from the Ophthalmology Department of Sichuan Provincial People’s Hospital. Patient selection was conducted by a single ophthalmologist, and the inclusion criteria were as follows: (1) Best-corrected visual acuity (BCVA) of less than 0.3 logMAR due to cataracts; (2) Individuals with cataracts whose visual function does not meet daily needs and who seek improvement in vision are expected to benefit from cataract extraction surgery, leading to enhanced visual function; (3) Regardless of gender or ethnicity. The exclusion criteria were: (1) Patients with a confirmed diagnosis of dementia; (2) Patients with other ocular diseases, excluding cataracts; (3) Patients with intraoperative or postoperative complications.

Unilateral cataract surgery was performed on patients with a best-corrected visual acuity (BCVA) of less than 0.3 logMAR in one eye. Bilateral cataract surgery was performed on patients with a BCVA of less than 0.3 logMAR in both eyes, or those whose binocular visual function was insufficient for daily activities. This classification resulted in two groups: the unilateral surgery group and the bilateral surgery group. Based on cognitive assessments conducted at enrollment, patients were classified into either the cognitively normal group or the mild cognitive impairment (MCI) group.

The study protocol complies with the ethical principles outlined in the Declaration of Helsinki and has received approval by the Ethics Committee of Sichuan Provincial People’s Hospital (No. Lunsen (Research) 2024 No. 312). Informed consent was obtained from all enrolled participants.

### 2.2 Research methods and outcome measures

The study design included four distinct time points: T0 (1 day preoperatively), T1 (1 week postoperatively), T2 (1 month postoperatively), and T3 (3 months postoperatively). At T0, all patients underwent routine preoperative assessments to exclude any surgical contraindications, as well as specialized ophthalmic evaluations, including slit-lamp examination of the anterior segment, BCVA, intraocular pressure measurement, anterior segment and fundus assessments, and visual and cognitive function testing. Follow-up assessments at T1, T2, and T3 evaluated BCVA (with the operated eye assessed in the unilateral group and the left eye in the bilateral group), as well as cognitive and visual function. Furthermore, at T2, a follow-up fundus examination was conducted to identify any previously undetected fundus diseases that may have been obscured by significant preoperative lens opacity. Visual acuity outcomes were converted to logMAR units, with counting fingers vision corresponding to 2.10 logMAR and light perception vision corresponding to 2.70 logMAR (Day et al., 2015).

All patients underwent micro-incision phacoemulsification cataract surgery, followed by intraocular lens implantation, with all procedures performed by the same surgeon.

Visual function assessments were conducted using a virtual reality (VR)-based binocular vision examination system, developed by the National Medical Device Engineering Technology Research Center, under 3D viewing conditions without glasses, to detect and quantify neural deficits in the visual pathways. The testing parameters included low spatial frequency suppression, simultaneous vision (both static and dynamic), and stereopsis (dynamic stereopsis, contour integration stereopsis, crowded stereopsis, color stereopsis, and red-blue line stereopsis), as well as perceptual eye position.

Cognitive function was assessed using the Montreal Cognitive Assessment (MoCA) scale. Participants with an educational background of 12 years or less received an additional point on their total score for the MoCA, and the higher scores indicate the better cognitive function. The cutoff points for grouping based on MoCA scores are as follows: individuals classified as illiterate scored 13/14, those with an educational background of 1 to 6 years scored 19/20, and those with 7 or more years of education scored 24/25 (Lu et al., 2011). These scores are used to determine inclusion in either the MCI group or the cognitively normal group.

### 2.3 Statistics

Statistical analyses were carried out using SPSS version 27.0. Descriptive statistics were generated for baseline characteristics as well as for preoperative and postoperative measurements of visual acuity, cognitive function, and individual visual function modules. Continuous variables are expressed as mean ± standard deviation (X ± SD) or median (Q1, Q3), while categorical variables were presented as frequency (n%). Intragroup comparisons were conducted using repeated measures ANOVA, the Friedman test, and the Cochran-Q test, with Bonferroni correction applied for *post-hoc* pairwise comparisons. Intergroup comparisons were made using the independent samples t-test, Mann–Whitney U test, and Fisher’s exact test. Statistical significance was set at *p* < 0.05.

## 3 Results

### 3.1 Demographic of the study population

A total of 35 cataract patients participated in this study, with a mean age of 69.9 ± 7.0 years. Among the participants, 13 were male (37.1%) and 22 were female (62.9%). The unilateral surgery group comprised 11 patients (31.4%), with a mean age of 72.0 ± 6.1 years, including 3 males (27.3%). The bilateral surgery group included 24 patients (68.6%), with a mean age of 69.0 ± 7.3 years, including 10 males (41.7%). The MCI group consisted of 25 patients (71.4%), with a mean age of 70.7 ± 7.4 years, including 8 males (32%). And the cognitively normal group included 10 patients (28.6%), with a mean age of 68.0 ± 5.7 years, including 5 males (50%). No statistically significant differences were observed in age and gender between the unilateral and bilateral surgery groups, as well as between the MCI and cognitively normal groups.

### 3.2 Visual acuity

**Figure 1:**
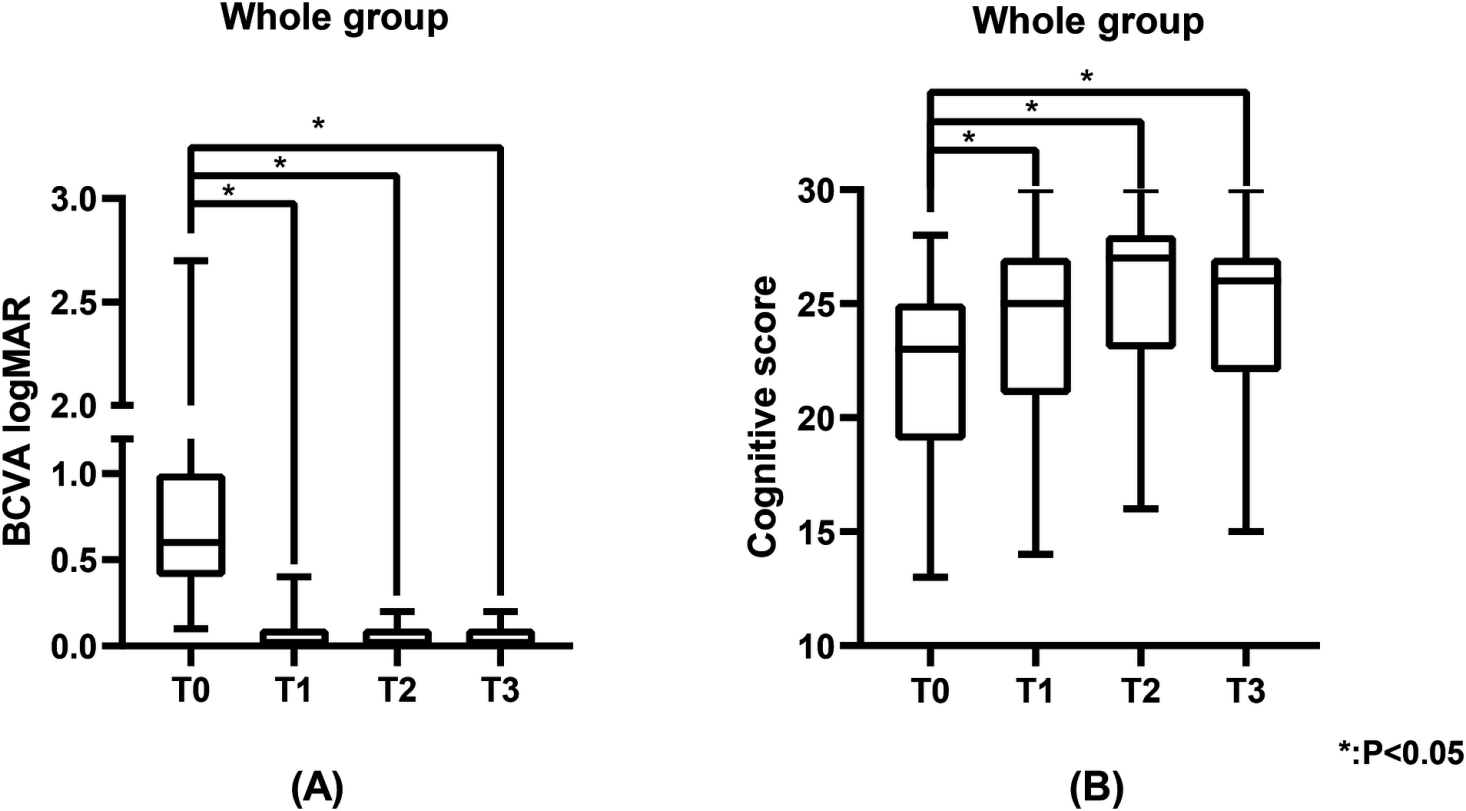
The BCVA logMAR and cognitive scores of the study population. **(A)** Represents the BCVA logMAR of the study population, and **(B)** represents the cognitive scores of the study population. BCVA was measured using the standard logarithmic visual acuity chart and converted to logMAR visual acuity. Cognitive scores were measured using the MoCA scale.

**Figure 2:**
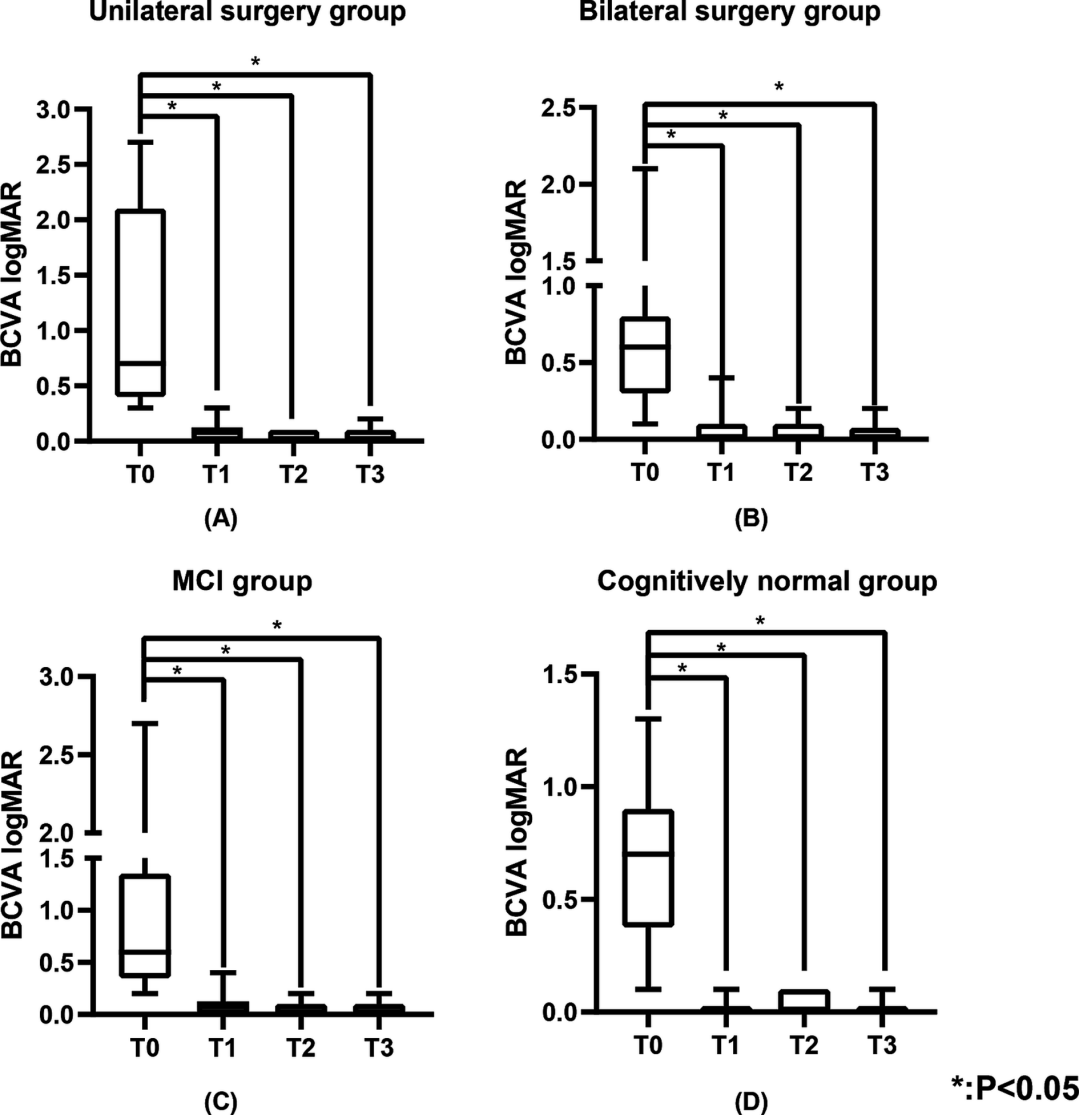
The BCVA logMAR of the unilateral surgery group, bilateral surgery group, MCI group, and cognitively normal group. **(A)** Represents the BCVA logMAR of the unilateral surgery group, **(B)** represents the BCVA logMAR of the bilateral surgery group, **(C)** represents the BCVA logMAR of the MCI group, and **(D)** represents the BCVA logMAR of the cognitively normal group. BCVA was measured using the standard logarithmic visual acuity chart and converted to logMAR visual acuity.

Table 1 shows the BCVA logMAR of the study population. The BCVA of all patients showed significant improvement at T1 (0.0 (0.0, 0.1) logMAR, *p* < 0.05), T2 (0.0 (0.0, 0.1) logMAR, *p* < 0.05), and T3 (0.0 (0.0, 0.1) logMAR, *p* < 0.05) compared to T0 (0.6 (0.4, 1.0) logMAR) (Figure 1A), with significant improvements observed in the unilateral surgery group, bilateral surgery group, MCI group, and cognitively normal group at the same time points (*p* < 0.05) (Figure 2). And no statistically significant differences in visual acuity were observed between the unilateral and bilateral surgery groups, or between the MCI and normal cognitive function groups at T0, T1, T2, and T3.

**Table 1:** The BCVA logMAR of the study population.

BCVA logMRA, X ± SD/M(Q1,Q3)	T0	T1	T2	T3	*p* value
All patients	0.6 (0.4,1.0)	0.0 (0.0,0.1)	0.0 (0.0,0.1)	0.0 (0.0,0.1)	<0.05^a,b,c^
Unilateral surgery group	0.7 (0.4,2.1)	0.1 (0.0,0.1)	0.0 (0.0,0.1)	0.0 (0.0,0.1)	<0.05^a,b,c^
Binocular surgery group	0.6 (0.3,0.8)	0.0 (0.0,0.1)	0.0 (0.0,0.1)	0.0 (0.0,0.1)	<0.05^a,b,c^
MCI group	0.6 (0.4,1.4)	0.1 (0.0,0.1)	0.0 (0.0,0.1)	0.0 (0.0,0.1)	<0.05^a,b,c^
Cognitively normal group	0.7 (±0.4)	0.0 (0.0,0.0)	0.0 (0.0,0.1)	0.0 (0.0,0.0)	<0.05^a,b,c^

T0, preoperative; T1, 1 week postoperative; T2, 1 month postoperative; T3, 3 months postoperative.

For *post hoc* tests, superscript a refers to significant difference between T0 and T1 (*p* < 0.05), superscript b refers to significant difference between T0 and T2 (*p* < 0.05) and superscript c refers to significant difference between T0 and T3 (*p* < 0.05).

### 3.3 Cognitive function

**Figure 3:**
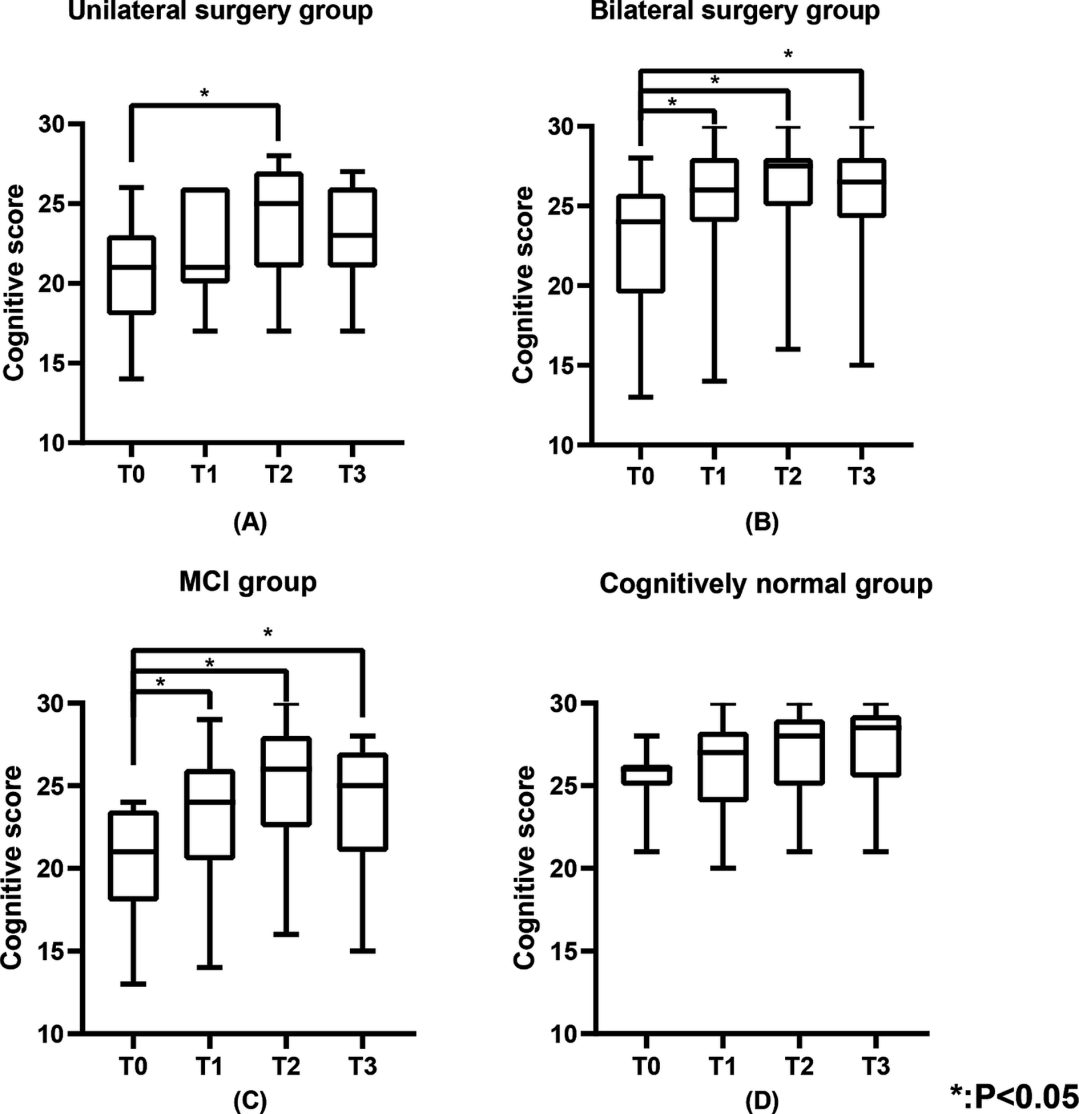
The cognitive scores of the unilateral surgery group, bilateral surgery group, MCI group, and cognitively normal group. **(A)** Represents the cognitive scores of the unilateral surgery group, **(B)** represents the cognitive scores of the bilateral surgery group, **(C)** represents the cognitive scores of the MCI group, and **(D)** represents the cognitive scores of the cognitively normal group.

Table 2 shows the cognitive function of the study population. Figure 3 shows the changes in cognitive scores for the four subgroups. Cognitive function demonstrated a significant improvement across the entire cohort at T1 (25.0 (21.0, 27.0), *p* < 0.05), T2 (27.0 (23.0, 28.0), *p* < 0.05), and T3 (26.0 (22.0, 27.0), *p* < 0.05) when compared to T0 (23.0 (19.0, 25.0)) (Figure 1B). In the unilateral surgery group, cognitive function demonstrated a significant improvement at T2 (23.6 (±3.7), *p* < 0.05) compared to T0 (20.2 (±3.9)). However, at T1 (21.8 (±3.0)) and T3 (22.8 (±3.2)), no statistically significant differences were observed when compared to T0. In the bilateral surgery group, cognitive function significantly improved at T1 (26.0 (24.0, 28.0), *p* < 0.05), T2 (27.5 (25.0, 28.0), *p* < 0.05), and T3 (26.5 (24.3, 28.0), *p* < 0.05) compared to T0 (24.0 (19.5, 25.8)). In addition, at T1, T2, and T3, cognitive scores in the bilateral surgery group were significantly higher than those in the unilateral surgery group (*p* < 0.05). In the MCI group, cognitive function demonstrated a significant improvement at T1 (23.1 (±4.2), *p* < 0.05), T2 (26.0 (22.5, 28.0), *p* < 0.05), and T3 (25.0 (21.0, 27.0), *p* < 0.05) compared to T0 (21.0 (18.0, 23.5)). In contrast, the cognitively normal group showed no statistically significant differences in cognitive function scores at T1 (26.3 (±3.0)), T2 (27.1 (±2.7)), and T3 (28.5 (25.5,29.3)) compared to T0 (26.0 (25.0,26.3)).

**Table 2:** The cognitive function of the study population.

Cognitive score, X ± SD/M(Q1,Q3)	T0	T1	T2	T3	*p* value
All patients	23.0 (19.0,25.0)	25.0 (21.0,27.0)	27.0 (23.0,28.0)	26.0 (22.0,27.0)	<0.05^a,b,c^
Unilateral surgery group	20.2 (±3.9)	21.8 (±3.0)	23.6 (±3.7)	22.8 (±3.2)	<0.05^b^
Binocular surgery group	24.0 (19.5,25.8)	26.0 (24.0,28.0)#	27.5 (25.0,28.0)#	26.5 (24.3,28.0)#	<0.05^a,b,c^
MCI group	21.0 (18.0,23.5)	23.1 (±4.2)	26.0 (22.5,28.0)	25.0 (21.0,27.0)	<0.05^a,b,c^
Cognitively normal group	26.0 (25.0,26.3)	26.3 (±3.0)	27.1 (±2.7)	28.5 (25.5,29.3)	–

T0, preoperative; T1, 1 week postoperative; T2, 1 month postoperative; T3, 3 months postoperative.

For *post hoc* tests, superscript a refers to significant difference between T0 and T1 (*p* < 0.05), superscript b refers to significant difference between T0 and T2 (*p* < 0.05) and superscript c refers to significant difference between T0 and T3 (*p* < 0.05).

Superscript # indicates that at T1, T2, and T3, the cognitive scores of the bilateral surgery group were significantly higher than those of the unilateral surgery group (*p* < 0.05).

### 3.4 Visual function

**Figure 4:**
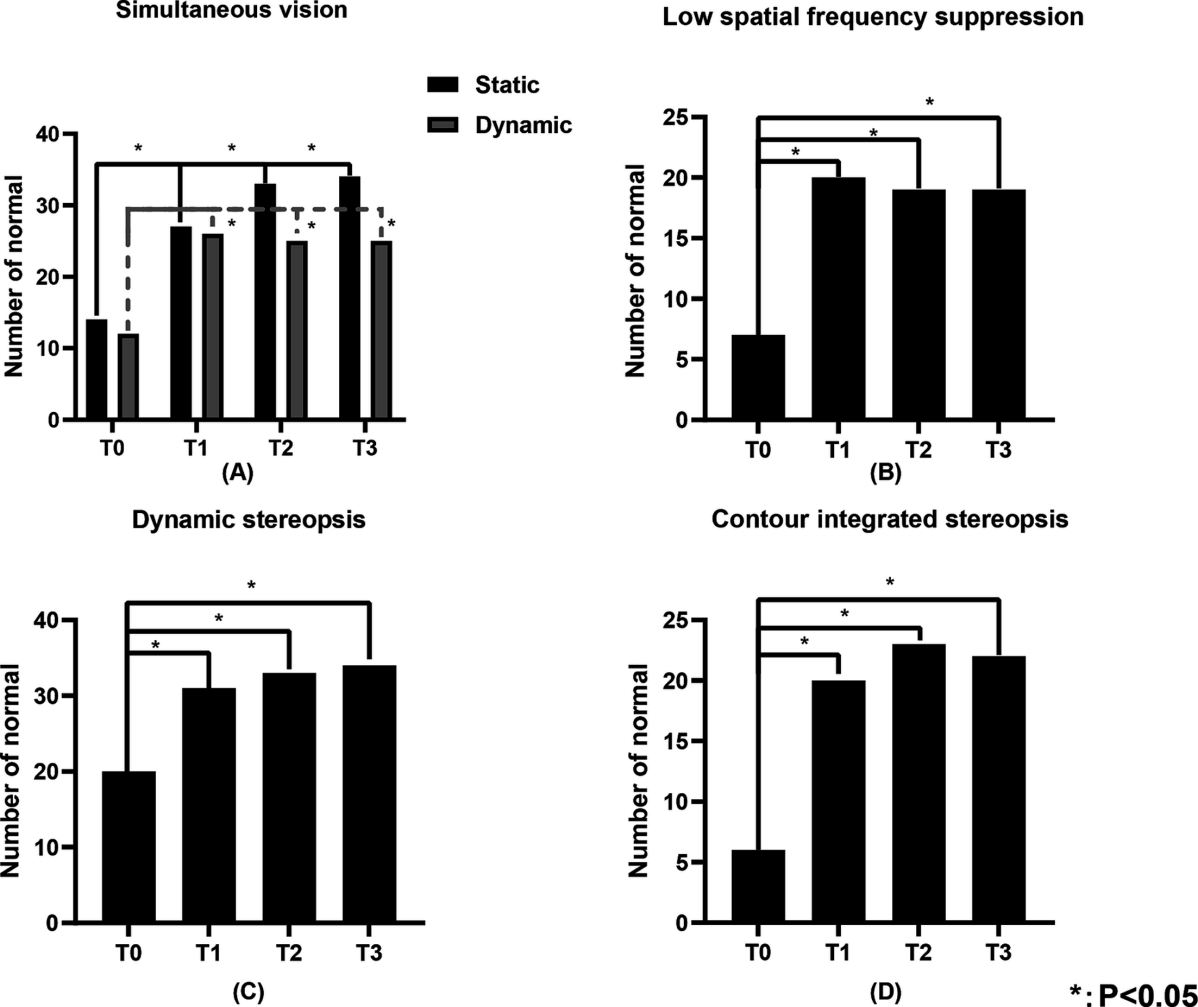
The number of individuals in the study population with normal static simultaneous vision, dynamic simultaneous vision, low spatial frequency suppression, dynamic stereopsis and contour integration stereopsis of the study population. **(A)** Represents the static simultaneous vision and dynamic simultaneous vision, **(B)** represents the low spatial frequency suppression, **(C)** represents the dynamic stereopsis, and **(D)** represents the contour integration stereopsis. “Number of normal” represents the number of individuals with normal visual function.

**Figure 5:**
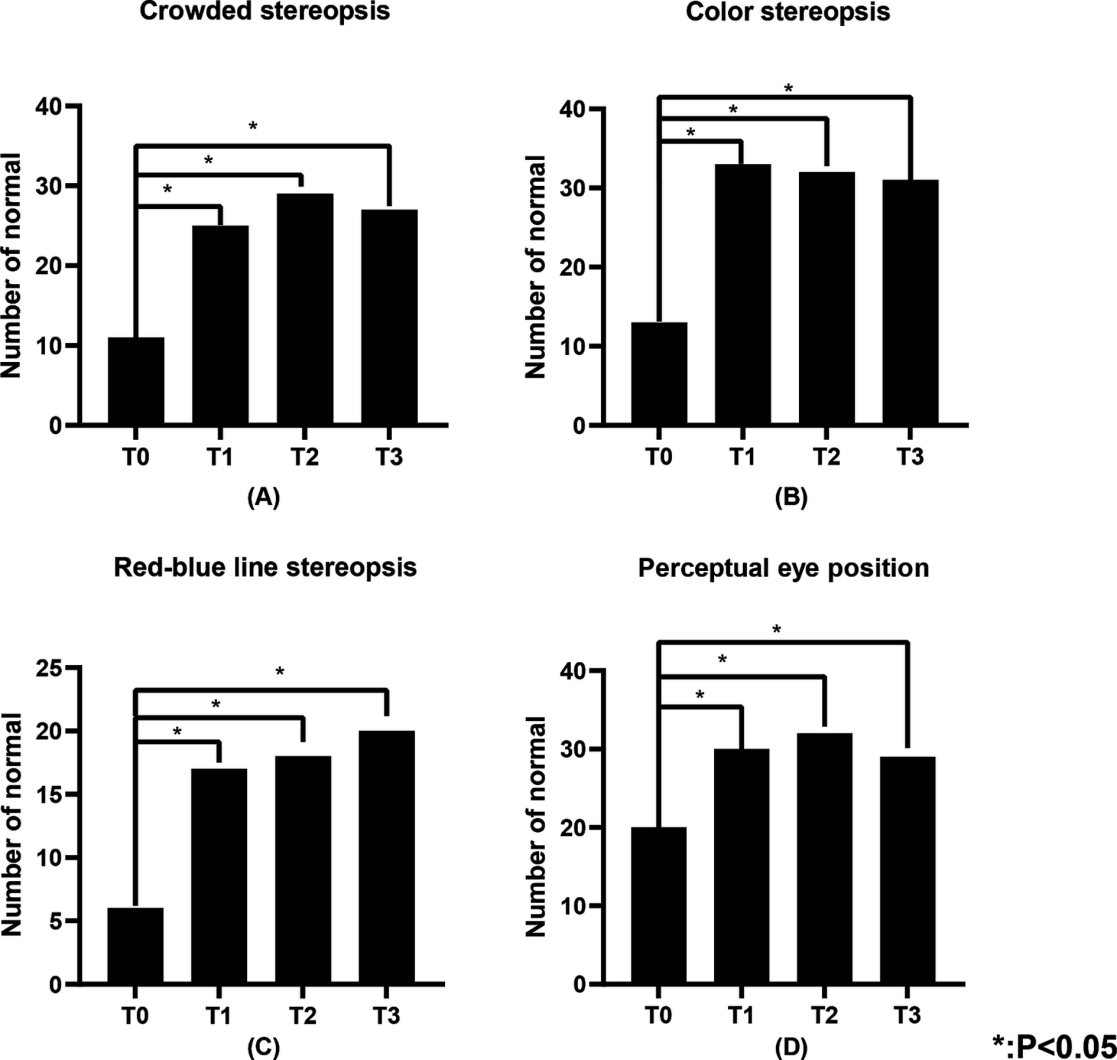
The number of individuals in the study population with normal crowded stereopsis, color stereopsis, red-blue line stereopsis, and perceptual eye position. **(A)** Represents the crowded stereopsis, **(B)** represents the color stereopsis, **(C)** represents the red-blue line stereopsis, and **(D)** represents the perceptual eye position. “Number of normal” represents the number of individuals with normal visual function.

Table 3 illustrates the number of individuals in the study population with normal visual function. Figures 4, 5 show the changes in visual function in the study population. At T0, the proportion of patients exhibiting normal visual function was relatively low, with the lowest rates of normalcy observed for contour integration stereopsis and red-blue line stereopsis (both 17.1%). Significant improvements in low spatial frequency suppression, static simultaneous vision, dynamic simultaneous vision, dynamic stereopsis, contour integration stereopsis, crowded stereopsis, color stereopsis, red-blue line stereopsis, and perceptual eye position were observed at T1, T2, and T3 compared to T0 (*p* < 0.05). Notably, dynamic stereopsis, crowded stereopsis, and color stereopsis exhibited the most substantial recovery compared to preoperative assessments. At the end of the follow-up, the normal rate of static simultaneous vision and dynamic stereopsis was highest (both 97.1%). Static simultaneous vision in the unilateral surgery group demonstrated significant improvement at T2 and T3 compared to T0 (*p* < 0.05). Patients in the bilateral surgery group exhibited significant improvements in low spatial frequency suppression, static simultaneous vision, dynamic simultaneous vision, dynamic stereopsis, contour integration stereopsis, crowded stereopsis, color stereopsis, and red-blue line stereopsis at T1, T2, and T3 compared to T0 (*p* < 0.05). The perceptual eye position exhibited significant improvement at T1 and T2 compared to T0 (*p* < 0.05).

**Table 3:** The number of individuals in the study population with normal visual function.

*N* (%)	T0	T1	T2	T3	*p* value
Low spatial frequency suppression					
All patients	7 (20%)	20 (57.1%)	19 (54.3%)	19 (54.3%)	<0.05^a,b,c^
Unilateral surgery group	4 (36.4%)	6 (54.5%)	5 (45.5%)	5 (45.5%)	–
Binocular surgery group	3 (12.5%)	14 (58.3%)	14 (58.3%)	14 (58.3%)	<0.05^a,b,c^
Static simultaneous vision					
All patients	14 (40.0%)	27 (77.1%)	33 (94.3%)	34 (97.1%)	<0.05^a,b,c^
Unilateral surgery group	5 (45.5%)	9 (81.8%)	11 (100%)	11 (100%)	<0.05^b,c^
Binocular surgery group	9 (37.5%)	18 (75%)	22 (91.7%)	23 (95.8%)	<0.05^a,b,c^
Dynamic simultaneous vision					
All patients	12 (34.3%)	26 (74.3%)	25 (71.4%)	25 (71.4%)	<0.05^a,b,c^
Unilateral surgery group	5 (45.5%)	8 (72.7%)	7 (63.6%)	6 (54.5%)	-
Binocular surgery group	7 (29.2%)	18 (75%)	18 (75%)	19 (79.2%)	<0.05^a,b,c^
Dynamic stereopsis					
All patients	20 (57.1%)	31 (88.6%)	33 (94.3%)	34 (97.1%)	<0.05^a,b,c^
Unilateral surgery group	7 (63.6%)	8 (72.7%)	9 (81.8%)	11 (100%)	-
Binocular surgery group	13 (54.2%)	23 (95.8%)	24 (100%)	23 (95.8%)	<0.05^a,b,c^
Contour integration stereopsis					
All patients	6 (17.1%)	20 (57.1%)	23 (65.7%)	22 (62.9%)	<0.05^a,b,c^
Unilateral surgery group	2 (18.2%)	4 (36.4%)	4 (36.4%)	5 (45.5%)	–
Binocular surgery group	4 (16.7%)	16 (66.7%)	19 (79.2%)	17 (70.8%)	<0.05^a,b,c^
Crowding stereopsis					
All patients	11 (31.4%)	25 (71.4%)	29 (82.9%)	27 (77.1%)	<0.05^a,b,c^
Unilateral surgery group	4 (36.4%)	7 (63.6%)	8 (72.7%)	7 (63.6%)	–
Binocular surgery group	7 (29.2%)	18 (75%)	21 (87.5%)	20 (83.3%)	<0.05^a,b,c^
Color stereopsis					
All patients	13 (37.1%)	33 (94.3%)	32 (91.4%)	31 (88.6%)	<0.05^a,b,c^
Unilateral surgery group	5 (45.5%)	9 (81.8%)	10 (90.9%)	8 (72.7%)	–
Binocular surgery group	8 (33.3%)	24 (100%)	22 (91.7%)	23 (95.8%)	<0.05^a,b,c^
Red-blue line stereopsis					
All patients	6 (17.1%)	17 (48.6%)	18 (51.4%)	20 (57.1%)	<0.05^a,b,c^
Unilateral surgery group	4 (36.4%)	5 (45.5%)	4 (36.4%)	5 (45.5%)	–
Binocular surgery group	2 (8.3%)	12 (50.0%)	14 (58.3%)	15 (62.5%)	<0.05^a,b,c^
Perceptual eye position					
All patients	20 (57.1%)	30 (85.7%)	32 (91.4%)	29 (82.9%)	<0.05^a,b,c^
Unilateral surgery group	5 (45.5%)	8 (72.7%)	10 (90.9%)	9 (81.8%)	–
Binocular surgery group	15 (62.5%)	22 (91.7%)	22 (91.7%)	20 (83.3%)	<0.05^a,b^

T0, preoperative; T1, 1 week postoperative; T2, 1 month postoperative; T3, 3 months postoperative.

For *post hoc* tests, superscript a refers to significant difference between T0 and T1 (*p* < 0.05), superscript b refers to significant difference between T0 and T2 (*p* < 0.05) and superscript c refers to significant difference between T0 and T3 (*p* < 0.05).

## 4 Discussion

This study revealed that postoperative visual acuity significantly improved in patients with age-related cataracts, along with enhancements in cognitive and visual functions. Patients undergoing unilateral cataract surgery demonstrated a significant recovery in cognitive function only at 1 month postoperatively, whereas those undergoing bilateral cataract surgery exhibited substantial recovery at all follow-up time points compared to preoperative levels, with the bilateral surgery group exhibiting superior cognitive performance relative to the unilateral group. Patients with MCI demonstrated a significant recovery in cognitive function during the postoperative follow-up period, whereas the cognitively normal group exhibited no statistically significant differences in cognitive function when comparing postoperative assessments to preoperative levels. Patients in the unilateral surgery group exhibited a significant recovery in static simultaneous vision at both 1 month and 3 months postoperatively compared to preoperative levels. In the bilateral surgery group, significant improvements were observed in low spatial frequency suppression, static simultaneous vision, dynamic simultaneous vision, dynamic stereopsis, contour integration stereopsis, crowded stereopsis, color stereopsis, and red-blue line stereopsis throughout the postoperative follow-up period compared to preoperative measurements. Additionally, perceptual eye position exhibited significant recovery at both 1 week and 1 month postoperatively.

The findings of this study align with previous research, demonstrating that postoperative cognitive recovery in patients with age-related cataracts further corroborates the positive impact of cataract surgery on the restoration of cognitive function (Verdina et al., 2022). Prior research have proposed several hypotheses regarding the mechanisms by which cataracts contribute to cognitive impairment. The sensory deprivation hypothesis suggests that sensory impairments may result in reduced peripheral stimulus input, potentially accelerating neurodegeneration or exacerbating its effects through cortical atrophy (Lee et al., 2022; Luo et al., 2018). Visual impairment may result in reduced social engagement, which are closely associated with cognitive decline (Livingston et al., 2020). Additionally, it negatively affects emotional well-being, contributing to anxiety, depression, and loneliness (Virgili et al., 2022; Frank et al., 2019; Tang et al., 2024; Hreha et al., 2024; Hashemi et al., 2024). Furthermore depression has been reported to be associated with cognitive impairment (Butters et al., 2004). The recovery of cognitive function following cataract surgery may be associated with enhanced clarity of the refractive system, which improves the stimulation of visual information to the brain. Moreover, improvements in visual acuity may facilitate daily activities, encouraging older adults to engage more actively in social interactions, enhance emotional well-being, and potentially further promote cognitive improvement (Maharani et al., 2018; Ishii et al., 2008; Fraser et al., 2013; Wang S. et al., 2024). Additionally, it may also be associated with increased postoperative blue light stimulation, which benefits the excitation of retinal ganglion cells, triggering widespread cortical activity in the brain, thereby potentially promoting improvements in cognitive function (Schmoll et al., 2011; Vandewalle et al., 2009).

Cognitive function improvement after unilateral cataract surgery was significant only at 1 month postoperatively. This may be attributed to the relatively small sample size in the unilateral group, which could have limited the statistical power to detect differences in cognitive changes at 1 week and 3 months post-surgery. The pattern of cognitive function changes following cataract surgery was similar for both unilateral and bilateral procedures, with cognitive performance at 1 month post-surgery surpassing that at 3 months, followed by 1 week post-surgery, and finally preoperative levels. The greatest cognitive improvement occurred at 1 month post-surgery, which we hypothesize may be due to practice effects. Repeated cognitive assessments over the month likely familiarized patients with the test content, thereby enhancing their performance (Granholm et al., 2010; Calamia et al., 2012). Cognitive function following unilateral cataract surgery was less improved compared to bilateral surgery, potentially due to residual lens opacity in the contralateral eye. In contrast, bilateral cataract surgery alleviated binocular visual impairment, thereby reducing the impact of sensory deprivation on the nervous system. Additionally, bilateral surgery significantly enhanced visual function, which alleviated difficulties in daily living and emotional issues, ultimately facilitating a more effective recovery of cognitive function. Butters et al. (2004) reported a link between depression and cognitive impairment (Butters et al., 2004), while Fraser et al. (2013) observed a slight improvement in depressive symptoms after unilateral surgery (Fraser et al., 2013). Consequently, the poorer cognitive outcomes after unilateral cataract surgery may also be attributed to this factor.

Yoshida et al. (2024) reported that cognitive function improved in patients with MCI after cataract surgery, but no significant improvement was observed in patients with dementia. They suggested that the improvement in cognitive function after cataract surgery may be related to the preoperative cognitive status (Yoshida et al., 2024). This study also identified a significant improvement in cognitive function among MCI patients postoperatively. Research by Sachdev et al. identified that favorable visual conditions are among the beneficial factors promoting cognitive recovery in MCI patients, as visual impairments may diminish activities that contribute to cognitive reserve, such as reading (Sachdev et al., 2013). Yeo et al. (2024) reported that cataract surgery was associated with a 4% improvement in short-term cognitive test scores among participants with normal cognition, which contrasts with our findings. This discrepancy may be attributed to the relatively small sample size of cognitively normal individuals in our study.

Traditional methods for assessing binocular visual function include simultaneous vision, fusion abilities, and stereopsis, typically employing tools such as synoptophore, Worth Four-Dot Test, and Titmus Stereotest. In this study, we employed a VR-based system to evaluate binocular visual function, including low spatial frequency suppression, simultaneous vision, stereopsis, and perceptual eye position under 3D viewing conditions without glasses. Simultaneous vision refers to the ability of both eyes to perceive images from either side concurrently, integrating them into a single visual representation in the brain. Stereopsis encompasses depth perception and the interpretation of the 3D structure of objects through binocular disparity (Blake and Wilson, 2011). Adequate visual acuity is essential for achieving both simultaneous vision and stereopsis, with the latter requiring higher visual standards, necessitating that both eyes have similar levels of acuity to accurately compare disparities and generate depth perception. Perceptual eye position is measured under conditions of binocular dissociation, reflecting the visual cortex’s ability to independently control eye positions and the deviation between perceived and actual locations (Guo-hong et al., 2014; Jie et al., 2014). Low spatial frequency suppression refers to the visual system’s reduced response to low spatial frequency information, which relates to coarser and more indistinct image features (Mucke et al., 2013).

Previous studies examining visual function following unilateral or bilateral cataract surgery have predominantly utilized tools such as the VF-9 scale, the Catquest-9SF questionnaire, and the National Eye Institute Visual Function Questionnaire (NEI-VFQ-25) to assess visual function (Liu et al., 2022; Seth et al., 2022; Tan et al., 2012). However, research on the impact of unilateral and bilateral cataract surgery on visual function using VR-based visual assessment systems is relatively limited. This study identified a high prevalence of visual function abnormalities prior to surgery in patients with bilateral age-related cataracts, characterized by low spatial frequency suppression, inadequate simultaneous vision and stereopsis, and deviations in perceptual eye position, with contour integration stereopsis and red-blue line stereopsis displaying the most pronounced deficits. However, postoperative assessments revealed a significant recovery of these visual functions, particularly in dynamic stereopsis, crowded stereopsis, and color stereopsis, which demonstrated the most significant improvements.

Preoperative visual function in patients with age-related cataracts is frequently suboptimal, potentially due to varying degrees of lens opacification in both eyes, resulting in a decline in visual input quality and an imbalance of information. Postoperatively, as visual acuity improves, visual input becomes more balanced and clear, allowing the brain to process visual information more effectively, thereby accounting for the observed enhancement in visual function. The changes in visual function before and after cataract surgery may also be related to neuroplasticity (Johnson and Cohen, 2023). Prolonged exposure to low-quality visual input prior to surgery can variably affect the brain’s sensitivity to visual information, resulting in various visual impairments. Conversely, postoperative clarity in visual conditions facilitates varying degrees of recovery in visual function. This study observed that the proportion of normal contour integration stereopsis and red-blue line stereopsis was lowest preoperatively, while postoperative recovery was most pronounced in dynamic stereopsis, crowded stereopsis, and color stereopsis. This finding indicates a substantial impact of cataracts on stereoptic function, likely due to stereopsis necessitates a minimum level of good vision as a prerequisite (Kwapiszeski et al., 1996).

Moreover, significant differences in the postoperative recovery of visual functions were observed between the unilateral and bilateral surgery groups. Castells et al. (2006) have found that, compared to unilateral surgery, bilateral cataract surgery results in a twofold improvement in stereopsis, likely due to the reduction in the disparity between the visual acuity of the two eyes following bilateral surgery (Castells et al., 2006), and this is consistent with the findings of the our study. We hypothesize that the superior performance in low spatial frequency suppression, simultaneous vision, and perceptual eye alignment following bilateral cataract surgery, compared to unilateral surgery, may be related to the reduction in the visual disparity between the two eyes. In contrast, although unilateral surgery enhances the visual quality of the operated eye, the remaining lens opacity in the contralateral eye creates asymmetry in visual information, posing challenges for the brain in processing these asymmetric inputs. Consequently, these visual functions exhibit less significant improvement. Furthermore, because stereopsis and simultaneous vision are highly dependent on the coordination of both eyes, the improvements following bilateral surgery are more pronounced.

Both groups demonstrated recovery in static simultaneous vision postoperatively. However, recovery of dynamic simultaneous vision was observed exclusively in the bilateral surgery group. This phenomenon may be attributed to the relatively simple processing requirements of static visual information, which allows for a degree of simultaneous vision even in the presence of visual input asymmetry between the two eyes. In contrast, under dynamic conditions, the visual system must continuously update and synchronize input information from both eyes. This synchronization becomes particularly critical when processing the speed, direction, and depth information of objects, as interocular coordination is essential (Davids et al., 2005).

Postoperative changes in perceptual eye position in the bilateral cataract surgery group were unstable, with significant improvements observed in the short term, but no substantial changes in the long term. This observation may be attributed to the brain’s neuroplasticity and neural adaptability (Gulyaeva, 2017; Benda, 2021). The significant postoperative improvement in visual quality likely facilitated rapid adjustments in binocular fusion by the brain, with perceptual eye position gradually stabilizing over time as the visual system adapted. However, this hypothesis requires validation through long-term follow-up observations. Furthermore, the influence of visual quality on perceptual eye position may be relatively minor, as preoperative perceptual eye position already demonstrated superior performance compared to other visual function indicators. Retinal correspondence likely remains the principal factor influencing perceptual eye position (Tan et al., 2020).

## 5 Limitations

This study has several limitations. First, the sample size is relatively small, with a limited number of participants undergoing unilateral cataract surgery. This may have led to an underestimation of the impact of unilateral cataract surgery on the restoration of cognitive and visual functions. Second, the follow-up period was only 3 months, reflecting short-term changes in cognitive and visual functions post-surgery. The lack of long-term follow-up data limits the interpretation of these findings. As recovery of cognitive and visual functions may be gradual, our results should be interpreted with caution. Third, cognitive function was assessed solely using the MoCA scale. Although this tool is highly sensitive and specific for detecting MCI, its scores can be influenced by patients’ cultural backgrounds. Previous studies have shown that individuals with lower educational levels tend to score lower on certain tasks, potentially leading to a misdiagnosis of cognitive impairment (Pinto et al., 2019). Thus, we may have underestimated the cognitive scores of the participants. Fourth, due to the many factors influencing cognitive function, this study did not adequately control for confounding variables such as age, sex, comorbidities, mental state, and medication history, all of which could affect cognitive outcomes. Despite these limitations, the strengths of this study include its longitudinal design, which examined the effects of unilateral and bilateral cataract surgery on cognitive and visual functions. These findings provide valuable insights for guiding future interventions aimed at optimizing the restoration of both visual and cognitive functions in patients.

## 6 Conclusion

This study demonstrates that cataract surgery not only significantly improves visual acuity but also enhances both cognitive and visual functions. Bilateral surgery, in particular, yields more pronounced improvements in cognitive and visual functions compared to unilateral surgery, suggesting it confers greater benefits for elderly cataract patients. Additionally, this study found that patients with MCI experience significant improvements in cognitive function following cataract surgery, suggesting that cataract surgery provides a favorable condition for the cognitive recovery of MCI patients. Based on these findings, we recommend bilateral cataract surgery for elderly patients, especially those with MCI, who should undergo surgery as early as possible.

## Data Availability

The raw data supporting the conclusions of this article will be made available by the authors, without undue reservation.
